# Massachusetts Veterinary Medical Association

**Published:** 1897-04

**Authors:** Henry S. Lewis

**Affiliations:** Secretary


### MASSACHUSETTS VETERINARY MEDICAL ASSOCIATION.

THE regular monthly meeting was held at No. 19 Boylston Place, Boston, on Wednesday evening, January 27, 1897. The President, John M. Parker, called the meeting to order at 8 P.M. Members present: Drs. Beckett, Blackwood, Burr, Cronan, Frothingham, Howard, Lee, Lewis, McLaughlin, Parker, Peters, Pierce, Rogers, Winslow, Winchester, Stickney.

Dr. McLaughlin reported the malformation of a tooth met with recently in practice. Dr. Winchester reported a case where he found adhesion of one testicle to the peritoneum. Dr. Cronan had a dog presented at a clinic with a half-dozen twists in the off forward leg. Dr. Beckett reported a case of "Bastard Strangles" which made a good recovery. Dr. Howard reported an interesting case of a pony which ran away and severed the jugular. When he saw, it some time after, a clot had formed, and it made a good recovery. Dr. Cronan reported the successful removal of a prolapsus uteri by ligature.

Meeting adjourned at 10.10 P.M. HENRY S. LEWIS,
Secretary.

